# Supplementary material for: Symptom-Based Predictive Model of COVID-19 Disease in Children
Source: Viruses. 2021 Dec 30;14(1):63. doi: 10.3390/v14010063 (PMC8779426; doi:10.3390/v14010063)
Supplement: Supplementary file 1 [file viruses-14-00063-s001.zip › viruses-1491528-supplementary material.pdf]

Supplementary Information

‡Members of the COPEDICAT Research group: Isabel Zambudio, Hospital de Igualada i Atenció Primària de l'Anoia; Zulema Lobato, Hospital Althaia Manresa; Núria López, Hospital Universitari del Mar; Ana Moreira, Fundació Hospital Sant Joan de Déu de Martorell; Evelyn Berbel Palau, Fundació Hospital Sant Joan de Déu de Martorell; Mayela Solis, Fundació Hospital Sant Joan de Déu de Martorell; Glòria Ruiz, Equip de Pediatria dels Pirineus, Alt Urgell i Pallars; Xavier Bruna, EAP Baix Berguedà; Neus Rius, Hospital Universitari San Joan de Reus; Marc García-Lorenzo, Hospital Universitari San Joan de Reus; Abel Martínez, Consorci Sanitari de Terrassa; Marina Fenoy, Consorci Sanitari de Terrassa; Emiliano Mora, Hospital Mútua de Terrassa; Blanca Rosich, Hospital Universitari Joan XXIII Tarragona; Arantxa Gómez, Hospital Universitari Joan XXIII Tarragona; Clara Calbet, Pius Hospital de Valls; Montse Ruiz García, Hospital General de Vic; Ana M Moreno, EAP Ripollet; Imma Bayona, EAP Ripollet; Carlos Losana, CAP Poblenou (Barcelona); Teresa Fenollosa, Gran Sol Badalona; Coral Moreno, Equip Territorial de Pediatria del Garraf; Joan Azemar, Equip Territorial de Pediatria del Garraf; Maria Esteller, CAP Aldea-Camarles-l'Ampolla; Silvia Burgaya, EAP Manlleu; Teresa Riera, EAP Vic Nord; Xavi Durán, EAP Vilablareix (Girona); Pili Villalobos, Hospital de Figueres (Alt Empordà); Anna Fàbrega, Alt Empordà (Primària); Almudena Sánchez, CAP Les Hortes; Neus Piqué Palacín, ABS Pla d´Urgell (Mollerussa); Mireia Carulla Bonjoch, ABS Pla d´Urgell (Mollerussa); Angels Naranjo, CAP Montblanc; Olga Salvado, CAP Llibertat Reus; Lorena Braviz, CAP Cambrils (Tarragona); Maria Chiné, CAP Almacelles; Mireia Biosca, ABS Les Borges Blanques; Evaristo Galdeano, ABS Cappont Lleida; Mercé Escuer Morell, CAP Onze de Setembre Lleida; Silvia Prado, ABS Eixample Lleida; Raquel Plasencia; ABS Rural Sud-Granadella (Lleida); Mercé Giribet Folch, ABS Bordeta-Magraners (Lleida); Rebecca Oglesby, CAP Sort/Hospital Pallars Lleida; Isabel Vives, Hospital QuirónSalud Barcelona; Anna Mª Ristol, CAP Can Serra Hospitalet de Llobregat; Vanessa Laveglia, Hospital Universitari General de Catalunya; Javier Cantero, Corporació del Maresme i la Selva; Laia Solé, EAP Salt; Iris González, Consorci Sanitari de Terrassa; Lidia Busquets, EAP Vic Sud; Lidia Aulet; EAP Vic Sud; Maria Mendoza, EAP Vic Sud; Ramona Martin, CAP Marià Fortuny Reus; Sandra Segura, CAP Montroig Tarragona; Gemma Ricós, CAP Drassanes; Esperança Macià, EAP Manlleu; Francesc Fornaguera, CAP La Garriga; Verónica Sandra López, CAP Centre L'Hospitalet del Llobregat; Sonia Asensio, CAP Hospitalet del Llobregat; Alicia Rodríguez, CAP Just Hospitalet Llobregat; Ana Maldonado, Centro Médico Teknon; Isabel Aguilar Moliner, ABS Sant Boi; Maria Luisa Villalobos, CAP Sabadell Nord; Sandra Pérez, EAP Barberà del Vallés; Raquel Mañas Lorente, EAP Sabadell Centre; Irene Marín, EAP Sabadell Centre; Gisela Roca, EAP Sabadell Centre; Maria Montserrat Liani, EAP Igualada Nord; Mireia Arnan, EAP Igualada Nord; Agustí Iglesias, CAP Camps Blancs ABS Sant Boi; Merce Grau, EAP Sant Quirze; Maria Coma, Hospital Universitari Joan XXIII de Tarragona; Miriam González Moreno, CAP Can Oriac Nord (Sabadell); Gabriela Quezada, CAP Marià Fortuny Reus (Tarragona).

Results of the χ^2-test

The results of the χ^2-test can be seen in Figure S1. Variables in green, below the mean incidence value, stand for features that correlate with a negative COVID-19 test result, while those in red are present in a child testing positive for COVID-19. Strong deviations from the mean incidence in one way or the other are equally important, as they indicate that the variable could be a potential predictor of COVID-19 in children.

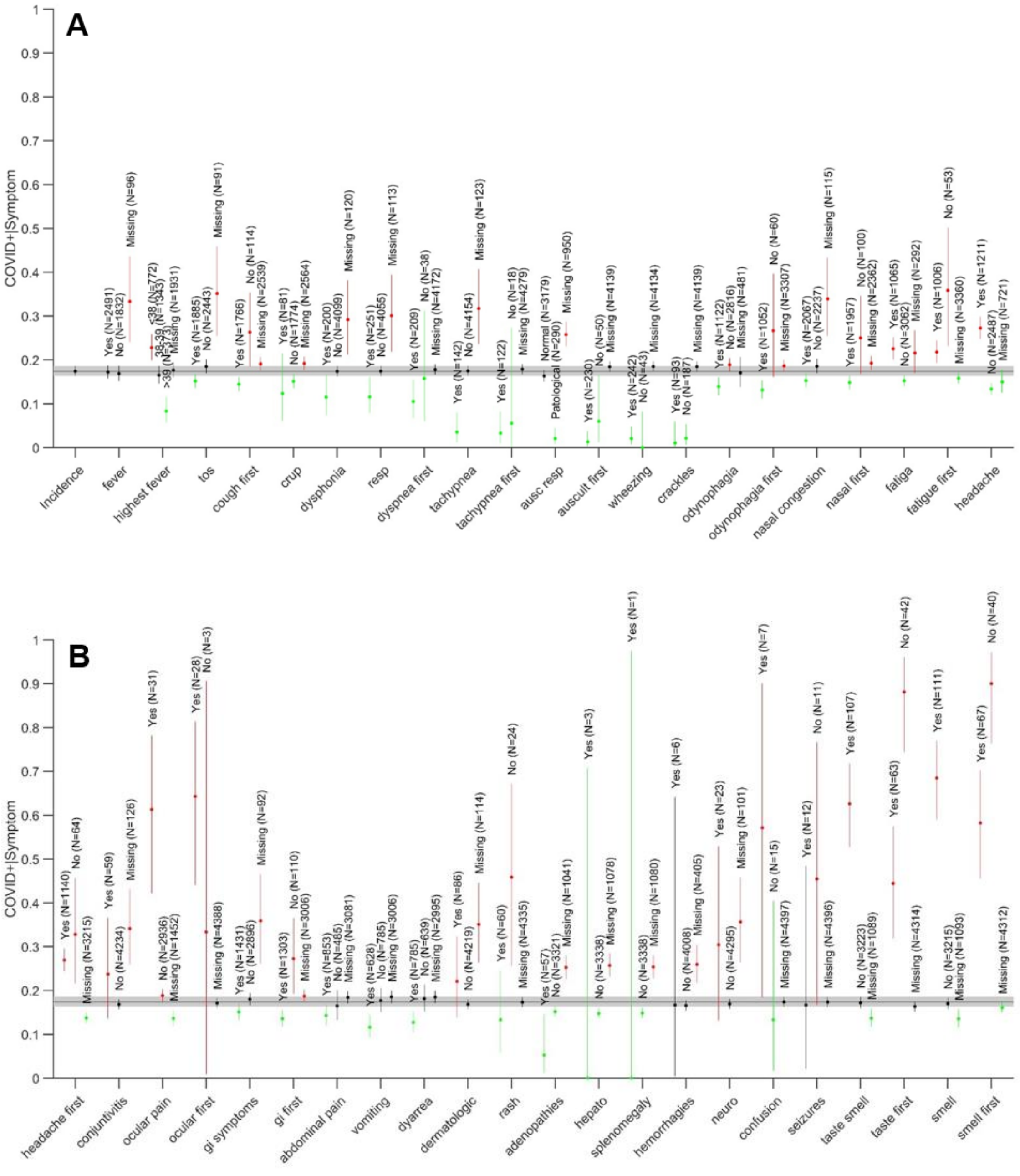

Figure S1. Incidence of a COVID-19 testing positive by RDT or PCR per significant symptom (P-value <.05 in the χ2-square test). We divided the symptoms in two different figures (A and B) to improve the visualization.

Description of the age-divided subsets used for training

Table S1. Specifications of the dataset used to train the predictive model of COVID-19 in patients aged 0 to 5 years old.

**Characteristic**	**Total**<br>**N (%)**	**Covid-19**<br>**N (%)**	**No covid-19**<br>**N (%)**
Fever			
No	95 (29.50)	52 (32.30)	43 (26.71)
37.5 ^{o}C to < 38 ^{o}C	55 (17.08)	41 (25.47)	14 (8.70)
38 ^{o}C to 39 ^{o}C	139 (43.17)	57 (35.40)	82 (50.93)
> 39 ^{o}C	33 (10.25)	11 (6.83)	22 (13.66)
Unknown	- (-)	- (-)	- (-)
Cough			
No	155 (48.14)	78 (48.45)	77 (47.83)
Yes	166 (51.55)	83 (51.55)	83 (51.55)
Unknown	1 (0.31)	0 (0)	1 (0.62)
Total days of fever			
None	118 (36.65)	65 (40.37)	53 (32.92)
1 or 2 days	147 (45.65)	74 (45.96)	73 (45.34)
3 to 7 days	55 (17.08)	20 (12.42)	35 (21.74)
>7 days	2 (0.62)	2 (1.24)	0 (0)
Unknown	- (-)	- (-)	- (-)
Auscultation			
Normal	242 (75.16)	119 (73.91)	123 (76.40)
Pathological Unknown	25 (7.76)	4 (2.48)	21 (13.04)
	55 (17.08)	38 (23.60)	17 (10.56)
Auscultation type			
Normal	297 (92.24)	157 (97.52)	140 (86.96)
Wheezing	14 (4.35)	3 (1.86)	11 (6.83)
Crackles	4 (1.24)	0 (0)	4 (2.48)
Both	7 (2.17)	1 (0.62)	6 (3.73)
Unknown	- (-)	- (-)	- (-)
Dysphonia			
No	303 (94.10)	151 (93.79)	152 (94.41)
Yes	17 (5.28)	9 (5.59)	8 (4.97)
Unknown	2 (0.62)	1 (0.62)	1 (0.62)
Respiratory sympt.			
No	304 (94.41)	156 (96.89)	148 (91.93)
Yes	17 (5.28)	5 (3.11)	12 (7.45)
Unknown	1 (0.31)	0 (0)	1 (0.62)
Tachypnoea			
No	304 (94.41)	156 (96.89)	148 (91.93)
Yes	14 (4.35)	4 (2.48)	10 (6.21)
Unknown	4 (1.24)	1 (0.62)	3 (1.86)
Odynophagia			
No	235 (72.98)	121 (75.16)	114 (70.81)
Yes	38 (11.80)	10 (6.21)	28 (17.39)
Unknown	49 (15.22)	30 (18.63)	19 (11.80)
Congestion			
No	141 (43.79)	69 (42.86)	72 (44.72)

Yes	177 (54.97)	92 (57.14)	85 (52.80)
Unknown	4 (1.24)	0 (0)	4 (2.48)
Fatigue			
No	256 (79.50)	125 (77.64)	131 (81.37)
Yes	40 (12.42)	21 (13.04)	19 (11.80)
Unknown	26 (8.07)	15 (9.32)	11 (6.83)
Headache			
No	203 (63.04)	92 (57.14)	111 (68.94)
Yes	31 (9.63)	22 (13.66)	9 (5.59)
Unknown	88 (27.33)	47 (29.19)	41 (25.47)
Conjunctivitis			
No	314 (97.52)	156 (96.89)	158 (98.14)
Yes	5 (1.55)	3 (1.86)	2 (1.24)
Unknown	3 (0.93)	2 (1.24)	1 (0.62)
Gastro sympt.			
No	237 (73.60)	111 (68.94)	126 (78.26)
Yes	85 (26.40)	50 (31.06)	35 (21.74)
Unknown	- (-)	- (-)	- (-)
Abdominal sympt.			
No	282 (87.58)	140 (86.96)	142 (88.20)
Yes	29 (9.01)	13 (8.07)	16 (9.94)
Unknown	11 (3.42)	8 (4.97)	3 (1.86)
Vomiting			
No	285 (88.51)	142 (88.20)	143 (88.82)
Yes	37 (11.49)	19 (11.80)	18 (11.18)
Unknown	- (-)	- (-)	- (-)
Diarrhoea			
No	274 (85.09)	131 (81.37)	143 (88.82)
Yes	48 (14.91)	30 (18.63)	18 (11.18)
Unknown	- (-)	- (-)	- (-)
Dermatologic			
No	314 (97.52)	155 (96.27)	159 (98.76)
Yes	7 (2.17)	5 (3.11)	2 (1.24)
Unknown	1 (0.31)	1 (0.62)	0 (0)
Rash			
No	319 (99.07)	160 (99.38)	159 (98.76)
Yes	3 (0.93)	1 (0.62)	2 (1.24)
Unknown	- (-)	- (-)	- (-)
Adenopathies			
No	259 (80.43)	125 (77.64)	134 (83.23)
Yes	2 (0.62)	0 (0)	2 (1.24)
Unknown	61 (18.94)	36 (22.36)	25 (15.53)
Haemorrhages			
No	297 (92.24)	146 (90.68)	151 (93.79)
Yes	- (-)	- (-)	- (-)
Unknown	25 (7.76)	15 (9.32)	10 (6.21)
Irritability			
No	190 (59.01)	101 (62.73)	89 (55.28)
Yes	27 (8.39)	16 (9.94)	11 (6.83)
Unknown	105 (32.61)	44 (27.33)	61 (37.89)

Neurological			
No	320 (99.38)	159 (98.76)	161 (100.00)
Yes	2 (0.62)	2 (1.24)	0 (0)
Unknown	- (-)	- (-)	- (-)
Shock			
No	314 (97.52)	157 (97.52)	157 (97.52)
Yes	- (-)	- (-)	- (-)
Unknown	8 (2.48)	4 (2.48)	4 (2.48)

Table S2. Specifications of the dataset used to train the predictive model of COVID-19 in patients aged 6 to 15 years old.

**Characteristic**	**Total N (%)**	**Covid-19 N (%)**	**No covid-19 N (%)**
Fever			
No	411 (54.22)	181 (47.76)	230 (60.69)
37.5 °C to < 38 °C	169 (22.30)	88 (23.22)	81 (21.37)
38 °C to 39 °C	156 (20.58)	98 (25.86)	58 (15.30)
> 39 °C	20 (2.64)	11 (2.90)	9 (2.37)
Unknown	2 (0.26)	1 (0.26)	1 (0.26)
Cough			
No	495 (65.30)	248 (65.44)	247 (65.17)
Yes	261 (34.43)	131 (34.56)	130 (34.30)
Unknown	2 (0.26)	0 (0)	2 (0.53)
Total days of fever			
None	463 (61.08)	207 (54.62)	256 (67.55)
1 or 2 days	232 (30.61)	131 (34.56)	101 (26.65)
3 to 7 days	53 (6.99)	34 (8.97)	19 (5.01)
>7 days	10 (1.32)	7 (1.85)	3 (0.79)
Unknown	- (-)	- (-)	- (-)
Auscultation			
Normal	511 (67.41)	254 (94.42)	257 (67.81)
Pathological	22 (2.90)	2 (0.74)	20 (5.28)
Unknown	225 (29.68)	13 (4.83)	102 (26.91)
Auscultation type			
Normal	736 (97.10)	377 (99.47)	359 (94.72)
Wheezing	20 (2.64)	2 (0.53)	18 (4.75)
Crackles	1 (0.13)	0 (0)	1 (0.26)
Both	1 (0.13)	0 (0)	1 (0.26)
Unknown	- (-)	- (-)	- (-)
Dysphonia			
No	735 (96.97)	370 (97.63)	365 (96.31)
Yes	18 (2.37)	6 (1.58)	12 (3.17)
Unknown	5 (0.66)	3 (0.79)	2 (0.53)
Respiratory sympt.			
No	719 (94.85)	361 (95.25)	358 (94.46)
Yes	35 (4.62)	16 (4.22)	19 (5.01)
Unknown	4 (0.53)	2 (0.53)	2 (0.53)
Tachypnoea			
No	740 (97.63)	375 (98.94)	365 (96.31)
Yes	10 (1.32)	0 (0)	10 (2.64)

Unknown	8 (1.06)	4 (1.06)	4 (1.06)
Odynophagia			
No	506 (66.75)	273 (72.03)	233 (61.48)
Yes	242 (31.93)	103 (27.18)	139 (36.68)
Unknown	10 (1.32)	3 (0.79)	7 (1.85)
Congestion			
No	463 (61.10)	236 (62.27)	227 (59.89)
Yes	285 (37.60)	139 (36.68)	146 (38.52)
Unknown	10 (1.32)	4 (1.06)	6 (1.58)
Fatigue			
No	479 (63.19)	233 (61.48)	246 (64.91)
Yes	272 (35.88)	143 (37.73)	129 (34.04)
Unknown	7 (0.92)	3 (0.79)	4 (1.06)
Headache			
No	371 (48.94)	164 (43.27)	207 (54.62)
Yes	377 (49.74)	212 (55.94)	165 (43.54)
Unknown	10 (1.32)	3 (0.79)	7 (1.85)
Conjunctivitis			
No	742 (97.89)	367 (96.83)	375 (98.94)
Yes	7 (0.92)	4 (1.06)	3 (0.79)
Unknown	9 (1.19)	8 (2.11)	1 (0.26)
Gastro sympt.			
No	488 (64.38)	270 (71.24)	218 (57.52)
Yes	269 (35.49)	108 (28.50)	161 (42.48)
Unknown	1 (0.13)	1 (0.26)	0 (0)
Abdominal sympt.			
No	559 (73.75)	305 (80.47)	254 (67.02)
Yes	198 (26.12)	74 (19.53)	124 (32.72)
Unknown	1 (0.13)	0 (0)	1 (0.26)
Vomiting			
No	650 (85.75)	340 (89.71)	310 (81.79)
Yes	108 (14.25)	39 (10.29)	69 (18.21)
Unknown	- (-)	- (-)	- (-)
Diarrhoea			
No	628 (82.85)	337 (88.92)	291 (76.78)
Yes	130 (17.15)	42 (11.08)	88 (23.22)
Unknown	- (-)	- (-)	- (-)
Dermatologic			
No	740 (97.63)	367 (96.83)	373 (98.42)
Yes	10 (1.32)	7 (1.85)	3 (0.79)
Unknown	8 (1.06)	5 (1.32)	3 (0.79)
Rash			
No	755 (99.60)	377 (99.47)	378 (99.74)
Yes	3 (0.40)	2 (0.53)	1 (0.26)
Unknown	- (-)	- (-)	- (-)
Adenopathies			
No	516 (68.07)	238 (62.80)	278 (73.35)
Yes	2 (0.26)	2 (0.53)	0 (0)
Unknown	240 (31.66)	139 (36.68)	101 (26.65)
Haemorrhages			

No	682 (89.97)	335 (88.39)	347 (91.56)
Yes	1 (0.13)	1 (0.26)	0 (0)
Unknown	75 (9.89)	43 (11.35)	32 (8.44)
Neurological			
No	751 (99.08)	375 (98.94)	376 (99.21)
Yes	5 (0.66)	3 (0.79)	2 (0.53)
Unknown	2 (0.26)	1 (0.26)	1 (0.26)
Shock			
No	717 (94.59)	363 (95.78)	354 (93.40)
Yes	- (-)	- (-)	- (-)
Unknown	41 (5.41)	16 (4.22)	25 (6.60)
Absence of taste			
No	678 (89.45)	325 (85.75)	353 (93.14)
Yes	53 (6.99)	49 (12.93)	4 (1.06)
Unknown	27 (3.56)	5 (1.32)	22 (5.80)
Absence of smell			
No	665 (87.73)	314 (82.85)	351 (92.61)
Yes	65 (8.58)	60 (15.83)	5 (1.32)
Unknown	28 (3.69)	5 (1.32)	23 (6.07)

Description of the modelling problem

For each age subset X_s, s {0, 1, 2}, we are given observations x_{is} with *j* symptomatology features. Each feature value x_{ijs} is categorical, and the task is to predict, given the ground truth labels y_{is} {0, 1}, the likelihood y_{is} of the observation x_{is} belonging to the class 1 or 0 (positive SARS-CoV-2 test or not).

Description of the solution implemented

We have built clinical predictive models f_s that issue the predictions $f_s(x_{is}) = y_{is}$. To capture interactions in the data, we have compared the performance of several machine learning models on each of the three datasets. The model that performed the best in each set was chosen.

Model architectures used

Boosted Trees

We are using the XGBClassifier implementation from XGBoost [1], which is a more regularized version of the Gradient Boosting Algorithm [2]. The XGBClassifier is an ensemble of decision trees, each of which is parametrized and sequentially improved via optimization algorithms. In particular, an XGB model is made up of classification and regression trees (CART) [3], that will provide the class into which the data sample is more likely to pertain. Each of these CARTs provides a prediction and then all of these are combined in order to form the final prediction for that data instance.

CART models are binary trees that have a root node, which receives a single feature of the input data and then has a split point regarding that feature. This split leads to an output, which is contained in a leaf and is then used to make a prediction. However, if there is more than one feature for the data instances, the leaf will actually turn into another split regarding one of the other features, which again will lead to another leaf that will contain an output. This process will continue until all features have been analysed and therefore, a prediction has been generated for that instance of data.

Each of the tree leaves will have an associated numerical score. This will help us to get not only the classification class but also the probability with which it was done. Then, each instance of data will get a score from each of the trees and in order to ensemble the data all of these will be added up and form the final score for that data instance.

Logistic Regression

Logistic regression [4] is a predictive analysis method that measures the relationship between a dichotomous or binary dependent variable and a set of independent variables by estimating probabilities using a logistic function, also known as sigmoid function, which is the cumulative distribution function of logistic distribution.

Unlike traditional regression [5], logistic regression does not attempt to predict a numeric variable's value from a set of inputs. Instead, assuming there are two classes, the output is the probability that the provided input point belongs to one of the classes. The logistic regression model does not perform statistical classification; however, it can be used to create a classifier by selecting a cut-off value and classifying inputs with probability greater than the cut-off as one class, and those with probability less than the cut-off as the other, resulting in a binary classifier. In other words, logistic regression is used to solve classification problems that require a probability estimate in the form of a 0/1.

Unlike linear regression [6], we do not fit a straight line to our data in logistic regression. Instead, we fit our data to a sigmoid curve, which is an S-shaped curve.

Logistic regression starts from a linear equation to predict the probability of presence of the characteristic of interest. However, this equation consists of log-odds which are further passed through a sigmoid function. This function, also called logistic function or transformations, takes any real input and squeezes the output of the linear equation to a probability between 0 and 1.

$$log\frac{p(x)}{1-p(x)} = \alpha_o + \alpha x \quad (1)$$

There are several methods to train the model. We can use an iterative optimization algorithm like gradient descent to calculate the parameters of the model (the weights) or we can use probabilistic methods like maximum likelihood.

Kernel Support Vector Machine (kSVM)

A Support Vector Machine (SVM) [7] is a supervised machine learning (ML) algorithm that can be employed for both classification and regression purposes. SVMs are more commonly used in binary classification problems. SVMs are based on the idea of finding a hyperplane that best and distinctly divides a dataset into N classes (**Figure S2**). At the end, hyperplanes are decision boundaries that help classify the data points depending on which side of it they fall.

For a classification task with only two features, a hyperplane is like a line that linearly separates and classifies a set of data. Intuitively, the further from the hyperplane our data points lie, the more confident we are that they have been correctly classified. We therefore want our data points to be as far away from the hyperplane as possible, while still being on the correct side of it. So, when new testing data is added, whatever side of the hyperplane it lands will decide the class that we assign to it.

The hyperplane function is defined as:

$$w^T x + b = 0 \quad (2)$$

where w is a weight vector, x is the input vector and b is the bias.

Another important concept is the margin of separation, which is the separation between the hyperplane and the closest data point. When we want to find the optimal hyperplane, what we do is to find the hyperplane for which the margin of separation is maximized. In the end, the problem of finding the

optimal hyperplane is an optimization problem, so we need training, and it can be solved by optimization techniques.
The support vectors are the data points that lie closest to the decision surface (or hyperplane), so they are the data points most difficult to classify and they have a direct impact on where the hyperplane should be placed.

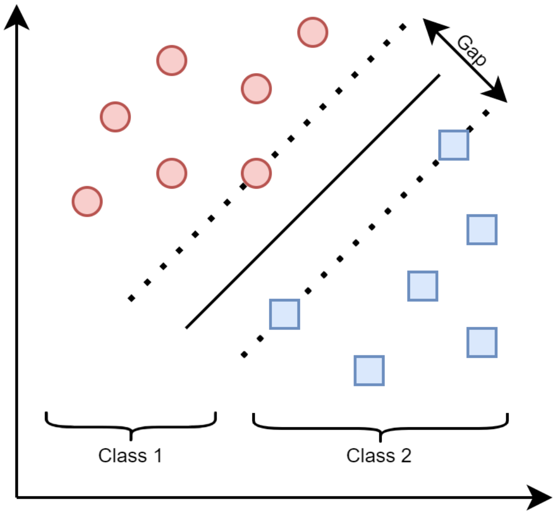

Figure S2. SVM hyperplane separating samples from two different classes

In the kernel version of this algorithm (kSVM) [7], which is the one used in the main article, the hyperplane is not computed on the original space, but rather in a transformed feature space, allowing for the separation of the data to be non-linear in the original space. This non-linearity is provided by the kernel function, which is used to map the observations from the original space to the transformed feature space. Which kernel function one picks is purely a design decision.

Multilayer Perceptron

The Multilayer Perceptron (MLP) [8] is an artificial neural network composed of input, hidden and output layers. Multiple hidden layers can be added to these networks to make them 'deep'. In principle, the more hidden layers, the bigger the abstraction capabilities of the model (limited by the data characteristics and amount). For this work, we have implemented a custom MLP class in the deep learning (DL) framework PyTorch [9] in order to optimize the hyperparameters as desired. This custom class follows the *scikit-learn* API, and one can easily specify the activation functions, the shape and quantity of hidden layers, the amount of dropout layers and dropout percentage.

Random Forest Classifier

As the name implies, a random forest (RF) [10] is made up of a large number of individual decision trees that work together as an ensemble. RFs create decision trees on randomly selected data samples, get a prediction from each tree and select the best solution by means of voting, the class with the most votes becomes the prediction of our model (**Figure S3**).
The wisdom of crowds is the basic principle behind RF, and it's a simple yet effective one. The RF model works so effectively because a large number of relatively uncorrelated models (trees) working together outperform any of the individual constituent models. The key is the low correlation between models. The explanation for this effect is that the trees protect each other from their individual mistakes (as long

as they don't all repeat the same errors). While some trees may be incorrect, many others will be correct, allowing the trees to move in the correct direction as a group.

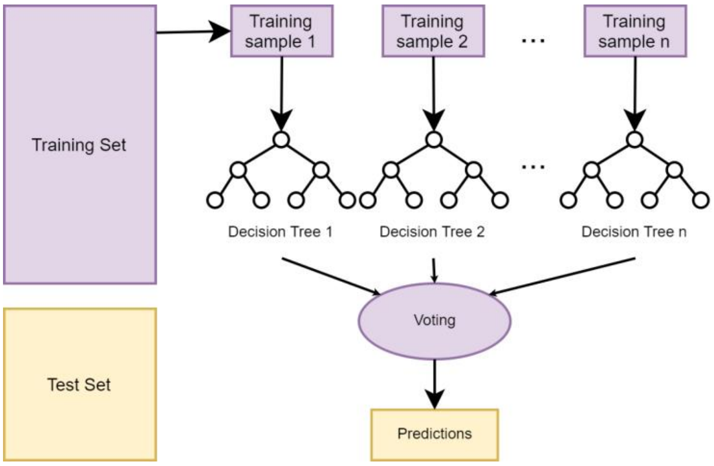

Figure S3. Visual description of how a Random Forest model provides a prediction

The method used by the RF to ensure that the behaviour of an individual decision tree does not correlate with the behaviour of the other trees is called bagging. Bagging is supported by the fact that decision trees are very sensitive to the data they are trained with. It works by allowing each individual tree to randomly sample from the dataset with replacement, resulting in different trees.
With bagging, we don't subset the training data into smaller chunks and train each tree on a different part. Rather, for each tree, we take a random sample of size N with replacement. To obtain the aggregation of the outputs of each simple and independent model, bagging uses the voting for the classification methods.

Dealing with missings

For the symptoms with less than 25% of missing values (the others were discarded in pre-processing), we have implemented an imputation routine. For this, *IterativeImputer* class from *scikit-learn* [11] has been used, which iteratively imputes each feature as a function of the others. The estimator used to model each feature as a function of the rest has been a k-nearest neighbours' classifier with Euclidean distance with the assumption of feature ordinality. We have set the number of rounds of the imputer to 3 and the number of neighbours for the estimator to consider (k) to 5.

Modelling pipeline

Model development
In order to provide a solution for our classification problem, we are using Machine and Deep Learning techniques, which means that our model will learn directly from data instead of predefining a descriptive equation. This modelling process mainly consists of two parts.
First, a training process, in which the model will use both the training features and the training labels in order to find the best model hyperparameters that allow a good fit between features and labels. In order to be able to declare which are these best hyperparameters, we need to have a way to measure their effectiveness, this is what we call the objective function, which will quantify how good the model fits by computing a training loss and adding a regularization factor. The training loss is a function that measures how well is our model doing with respect to the training data, that is, it computes the hit rate

of the model. However, if we only used the training loss to adjust our model, we would end up having a too specific fit that would only work for the training dataset and would not generalize properly for any other set of data. In order to keep the model from becoming too specific, what we call overfitting, the regularization term is added to the training loss. Therefore, one can see that there happens a trade-off between having a good yet simple model. This is commonly referred to as the bias-variance trade-off.

We split each data subset X_s into train and test in 70/30 proportions. Then, 5x2-fold cross validation (CV) is performed. In each CV fold the missing values in training and validation are imputed in a round-robin fashion for 5 rounds with a k-nearest-neighbour classifier (k=5). To prevent information leakage from sets when imputing, we first impute the training fold and then the validation fold, this time fitting the imputer both with the imputed training fold as well as the validation fold, so as to imitate the process of incorporating past information (as one would do in a real setting). Then, each feature is mapped into a one-hot representation, creating *n-1* columns for each feature if it originally had *n* values, and the training fold is undersampled such that there are 50% positive and 50% negative observations. With this transformed training data, we train the candidate model architectures with all of the chosen hyperparameter configurations and test their performance against the validation set. The performance is quantified in terms of area under the receiver operating characteristic curve (AUROC) curve, sensitivity, specificity, precision and F1 score. [12]

Model selection

We choose one hyperparameter configuration for any candidate model architecture: pairwise t-tests (in combination with 5x2 CV, as proposed in [13,14]) were performed to choose between hyperparameter configurations. Thus, we compared each of the AUC validation score sets obtained in the model development stage (10 scores for each model configuration) to see if they were statistically different from each other ($P<.05$) and finally we chose the hyperparameter configuration with the best average. See **Table S3** for an overview of the ranges of hyperparameters explored and **Table S5A** for the number of configurations tested.

Table S3. Grids of hyperparameter values explored in the model selection and fine-tuning steps of the modelling pipeline. Each hyperparameter configuration is created by randomly picking a hyperparameter value from the grid. [a-d]

Model type	Hyperparameter name	Possible values
**XGB**		
	learning rate	[0.001, 0.01, 0.1]
	n_estimators	[100, 300, 500]
	max_depth	[4, 6, 8]
	subsample	[0.25, 0.5, 0.8]
	gamma	[0, 0.1, 1]
	reg_alpha	[0, 0.1, 1]
	reg_lambda	[0, 0.1, 1]
**RF**		
	n_estimators	[25, 50, 100, 200, 300, 400, 500, 750, 1000]

max_depth	[3, 4, 5, 6, 7, 8, 9]
min_samples_split	[2, 4, 8, 16]
max_features	['auto', 'sqrt', 'log']
**MLP**	
linearLayerSizes	[((1.5, 2), (2, 1.5)), ((1.5, 2), (2, 3), (3, 2), (2, 1), (1, 0.5)), ((1.5, 2), (2, 3), (3, 3.5), (3.5, 3), (3, 2), (2, 1), (1, 0.5)), ((1.5, 2), (2, 3), (3, 4), (4, 3), (3, 2), (2, 1), (1, 0.5))]
activation	['relu', 'rrelu', 'tanh', 'sigmoid']
nDropoutLayers	[0.1, 0.2, 0.3]
dropoutP	[0.1, 0.15, 0.2]
nEpochs	[25, 50, 75, 100]
batchSize	[50]
initLR	[0.001]
**kSVM**	
C	[0.001, 0.005, 0.01, 0.05, 0.1, 1, 5, 10]
kernel	['poly', 'rbf', 'sigmoid']
degree	[2, 3, 4, 5, 6]
**LR**	
C	[0.0001, 0.0005, 0.001, 0.005, 0.01, 0.05, 0.1, 0.5, 1, 5, 10]

[a] linearLayerSizes: input and output sizes of the intermediate hidden layers as multipliers of the number of features in the data, in order. The first and last layers are implicitly created.
[b] activation: activation function used in hidden layers.
[c] nDropoutLayers: percentage of hidden layers that will be followed by a dropout layer.
[d] dropoutP: percentage of dropout in the dropout layers.

Table S4. (**A**) Number of hyperparameter configurations used in the model selection step of the pipeline. Each of the configurations for all the architectures is tried out for all three data subsets. (**B**) Number of hyperparameter configurations used in the fine-tuning step of the pipeline. Only one architecture is explored for each data subset. For both steps, all hyperparameter configurations are distinct.

**Architecture**	**XGB**	**RF**	**MLP**	**kSVM**	**LR**
**Nº configurations explored (model selection)**	120	120	120	120	11

(A)

**Architecture**	**XBG (All ages)**	**RF (0-5y)**	**RF (6-14y)**

**Nº configurations explored (fine-tuning)**	2187	756	756

(B)

Fine-tuning

With the best-performing classifier found in the model selection stage, we performed a more fine-grained hyperparameter search by repeating the model development stage but with more candidate configurations. The number of configurations used can be found in **Table S5B**.

Model evaluation

Each of the obtained model configurations in the model selection stage was trained with the full train set and bootstrap-evaluated against the test set. The full train set and the test set were previously transformed the same way as the train and validation sets in the model development stage: imputation, one-hot encoding and undersampling (only for training). 100 bootstrap rounds were performed to obtain 95% confidence intervals around each of the evaluation metrics.

Evaluation metrics

We tested the model's performance using the sensitivity, specificity, precision and F1 score metrics as well as the Area Under the Receiver Operating Characteristic (AUROC). They are defined as follows.

- Confusion Matrix

  As the name may hint, although the Confusion Matrix is not exactly an evaluation metric, it does allow one to see whether a model is confusing two classes and, furthermore, it determines the concepts that are then used to define the actual testing metrics. Thus, this table typically shows the number of samples from each class that have been respectively classified into one class (i.e., the correct one) or another (i.e., the incorrect one). This is, it will show the number of positive samples that have been classified as positive, those are the True Positive (TP), and also the positive samples that have been classified as negative, those are the False Negative (FN). Consequently, the same classification occurs for the negative samples; the ones classified as positive are the False Positive (FP), and the ones classified as negative are the True Negative (TN). Then, in general terms, **Figure S4** shows what a Confusion Matrix will look like.

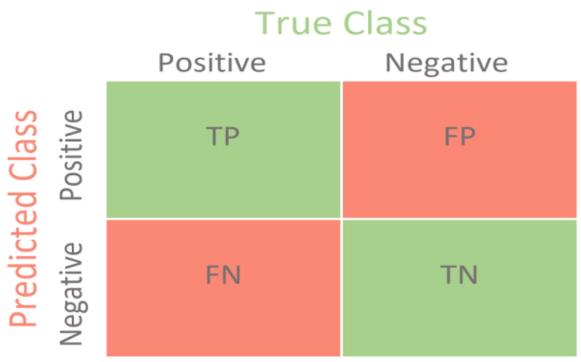

Figure S4. General representation of a Confusion Matrix [15]

- Sensitivity

  This metric, also known as Recall, mathematically describes the proportion of samples that were classified as positive by the model out of all the samples that were actually positive. That is, $\frac{TP}{TP+FN}$.

- Specificity

  This metric mathematically describes the proportion of samples that were classified as negative by the model out of all the samples that were actually negative. That is, $\frac{TN}{TN+FP}$.

- Precision

  This metric mathematically describes the proportion of samples that were classified as positive and were actually positive out of all the samples that were classified as positive by the model. That is, $\frac{TP}{TP+FP}$.

- F1 score

  This metric is actually the weighted average between the Recall, or Sensitivity, and the Precision. Therefore, it takes into account both False Negatives and False Positives, what makes this metric a more accurate one than traditional Accuracy (which is simply the ratio of correct predictions), especially for uneven class distributions. This is, $\frac{2\cdot(Recall\cdot Precision)}{Recall+Precision}$.

- AUROC

  The Area Under the Receiver Operating Characteristic (AUROC) is an evaluation metric for classification models [16], it then determines the ability of the model to classify a sample of a given class into that class. In our case, that is, the probability that the model will rank a uniformly drawn randomly selected patient that is Covid-19 positive higher than a uniformly drawn randomly selected patient that is Covid-19 negative. In other words, the area under the Receiver Operating Characteristic (ROC) curve is created by plotting the True Positive rate, also known as *Sensitivity* or *Recall*, against the False Positive rate, which is equivalent to *1-Specificity*, at various thresholds, as shown in **Figure S5**.

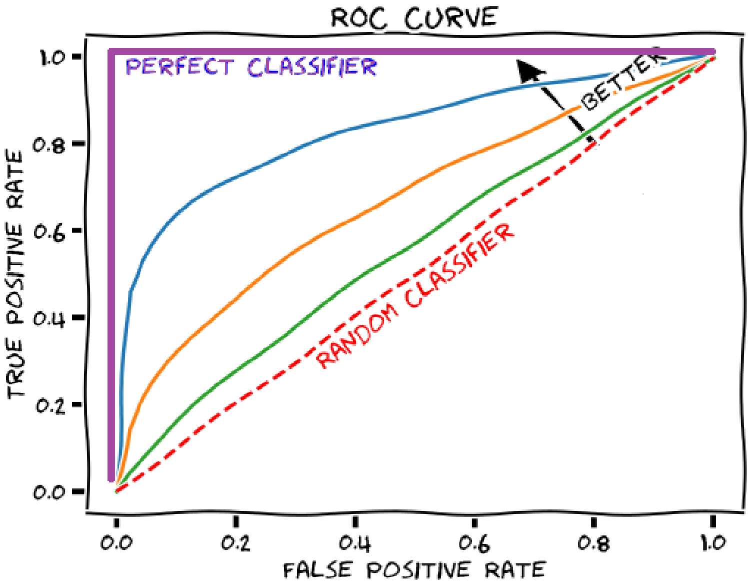

Figure S5. Visual description of the ROC curve [17].

Feature importance extraction

SHAP is a model-agnostic explainer method capable of providing additive feature importance values for any model's prediction, regardless of its architecture.

While we could have only interpreted the model trained with data from all age groups and then analysed the SHAP values of observations by different age strata instead of creating a classifier for each age interval, we believed the latter yields better interpretation. That is, in the first case, the SHAP values of observations in one age strata may possibly be influenced by observations of other age groups, having biased results depending on what the classifier really learned. On the other hand, having separate models ensures that the feature importance values of each age strata are not influenced by leading factors in other age groups. Given the models that have been chosen to perform best on the test data, we get the SHAP feature importance explanations for each of the test instances. That is, for each instance, we get a vector containing an approximation of the shapley value for each feature that tells us whether it affected positively or negatively the predicted probability of that test instance belonging to an infected individual or not.

In our case, we consider the shapley values with respect to the probability of being infected. With these local feature importance explanations, we average the shapley values along all of the test instances and obtain, for each feature, a mean shapley value. If that value is positive, it means that the feature has been, in most cases, a positive predictor of infection. On the other hand, if the value is negative, a high value of the feature (if the category is present) will, on average, push the predicted infection probability lower. With this procedure, we can not only obtain a measurement of importance of each feature for each of the three models, but also if the found relationship is positive or negative. So, we are able to see how the predictors change over the different age ranges.

Computational resources

Computational resources used in the pre-processing and modelling phases of our analysis can be found on **Table S5**.

Table S5. Computational resources used in the pre-processing and modelling phases and their IDs.

Resource	Source	ID
**Python**	python.org	3.7.11
**Python packages**		
scikit-learn	scikit-learn.org	0.24.2
PyTorch	pytorch.org	1.9.0+cu102
SHAP	github.com/slundberg/shap	0.39.0
pandas	pandas.pydata.org	1.1.5
numpy	numpy.org	1.19.5
**Source code**		
Data Processing, Model Generation, Evaluation, Importance Analysis	https://github.com/chus-chus/cov19-modeling	-

References

1. XGBoost: eXtreme Gradient Boosting. Available online: https://github.com/dmlc/xgboost (accessed on 15 October 2021).
2. Friedman, J.H. Greedy function approximation: A gradient boosting machine. *Ann. Statist.* **2001**, *29*, 1189–1232. https://doi.org/10.1214/aos/1013203451.
3. Krzywinski, M.; Altman, N. Classification and regression trees. *Nat. Methods* **2017**; *14*, 757–758. https://doi.org/10.1038/nmeth.4370.
4. Sperandei, S. Understanding logistic regression analysis. *Biochem. Med*. **2014**, *24*, 12–18. Available online: https://www.ncbi.nlm.nih.gov/pmc/articles/PMC3936971/ (accessed on 7 November 2021).
5. Weisberg, S. Regression Analysis. In *Encyclopedia of Computer Science and Engineering*; Wah, B.W.; John Wiley & Sons, Inc.: Hoboken, NJ, USA; 2007; https://doi.org/10.1002/9780470050118.ecse349.
6. Su, X.; Yan, X.; Tsai, C.-L. Linear regression. *WIREs Comp. Stat.* **2012**, *4*, 275–294. https://doi.org/10.1002/wics.1198.
7. Howley, T.; Madden, M.G. The Genetic Kernel Support Vector Machine: Description and Evaluation. *Artif. Intell. Rev.* **2005**, *24*, 379–395. https://doi.org/10.1007/S10462-005-9009-3.
8. Almeida, L.B. Multilayer perceptrons. In *Handbook of Neural Computation*; IOP Publishing Ltd and Oxford University Press in Westleigh, England, UK; 1997. Available online: http://www.lx.it.pt/~lbalmeida/papers/AlmeidaHNC.pdf (accessed on 7 November 2021).
9. Stevens, E.; Antiga, L.; Viehmann, T. Deep Learning with PyTorch, 1st ed.; *Manning Publications Co.*: Shelter Island, NY, USA; 2020.
10. Schonlau, M.; Zou, R.Y. The random forest algorithm for statistical learning. *Stata J.* **2020**, *20*, 3–29. https://doi.org/10.1177/1536867X20909688.
11. Pedregosa, F.; Varoquaux, G.; Gramfort, A.; Michel, V.; Thirion, B.; Grisel, O.; Blondel, M.; Prettenhofer, P.; Weiss, R.; Dubourg, V.; et al. Scikit-learn: Machine learning in Python. *J. Mach. Learn. Res.* **2011**, *12*, 2825–2830.
12. Dietterich, T.G. Approximate Statistical Tests for Comparing Supervised Classification Learning Algorithms. *Neural Comput*. **1998**, *10*, 1895–1923. https://doi.org/10.1162/089976698300017197.
13. Paszke, A.; Gross, S.; Massa, F.; Lerer, A.; Bradbury, J.; Chanan, G.; Killeen, T.; Lin, Z.; Gimelshein, N.; Antiga, L.; et al. PyTorch: An Imperative Style, High-Performance Deep Learning Library. In

Advances in Neural Information Processing Systems 32 [Internet]; Curran Associates, Inc.; Red Hook, NY, USA; 2019; pp. 8024–8035.
14. Lundberg, S.M.; Nair, B.; Vavilala, M.S.; Horibe, M.; Eisses, M.J.; Adams, T.; Liston, D.E.; Low, D.K.W.; Newman, S.F.; Kim, J.; et al. Explainable machine-learning predictions for the prevention of hypoxaemia during surgery. *Nat. Biomed. Eng.* **2018**, *2*, 749–760.
15. Confusion Matrix for Your Multi-Class Machine Learning Model. Available online: https://towardsdatascience.com/confusion-matrix-for-your-multi-class-machine-learning-model-ff9aa3bf7826 (accessed on 16 October 2021).
16. Hajian-Tilaki, K. Receiver Operating Characteristic (ROC) Curve Analysis for Medical Diagnostic Test Evaluation. *Casp. J Intern Med*. **2013**, *4*, 627–635.
17. Measuring Performance: AUC (AUROC). Available online: https://glassboxmedicine.com/2019/02/23/measuring-performance-auc-auroc/ (accessed on 16 October 2021).
